# Editorial: Exerscience: exploring physical activity’s role in diabetes and its complications

**DOI:** 10.3389/fendo.2026.1927361

**Published:** 2026-07-14

**Authors:** Shanhu Qiu, Evelyn B. Parr, Tongzhi Wu

**Affiliations:** 1 Department of General Practice, Zhongda Hospital, Institute of Diabetes, School of Medicine, Southeast University, Nanjing, China; 2 Research and Education Centre of General Practice, Zhongda Hospital, Southeast University, Nanjing, China; 3 Exercise and Nutrition Research Program, Mary MacKillop Institute for Health Research, Australian Catholic University, Fitzroy, VIC, Australia; 4 School of Medicine and Centre of Research in Translating Nutritional Science to Good Health, Adelaide University, Adelaide, SA, Australia

**Keywords:** complication, diabetes, endothelial function, exercise, physical activity

Regular exercise is integral to the prevention and management of diabetes. In addition to improving glycemic control, regular exercise favorably modifies cardiovascular risk factors, including blood pressure and body weight, and reduces the risk of both macrovascular and microvascular complications associated with diabetes (1). The articles included in this Research Topic have collectively reinforced the pivotal role of exercise in diabetes care, while highlighting emerging concepts and further research priorities.

High-intensity interval training (HIIT), characterized by repeated short bursts of aerobic exercise at vigorous-intensity interspersed with periods of brief recovery (e.g., low-intensity exercise), has attracted increasing attention as an efficient exercise strategy over the past two decades (2). Two complementary meta-analyzes of data from randomized trials by Li et al. added important insights into the metabolic benefits of HIIT. In individuals with prediabetes, HIIT was more effective than moderate-intensity continuous training (MICT) in lowering 2-hour plasma glucose, whereas MICT produced greater reductions in fasting plasma glucose. However, no significant differences were observed in hemoglobin A1c or lipid profiles between the two exercise interventions. In individuals with type 2 diabetes, compared with MICT, HIIT was associated with lower 24-hour mean glucose concentrations and less time spent in hyperglycemia as assessed by continuous glucose monitoring, although mean amplitude of glycemic excursions was comparable between these two interventions. Together, these findings suggest that HIIT may offer particular advantages for improving postprandial glycemic control. However, the trials included in both meta-analyzes were of small sample sizes (less than 50) and short term (less than 12 weeks of intervention). Whether these metabolic benefits translate into better long-term glycemic control and improved clinical outcomes in real-world practice remains to be established through larger, longer-term randomized trials among individuals with type 2 diabetes.

Accumulating evidence recommends combing aerobic exercise with resistance exercise to maximize metabolic benefits. Previous studies have also demonstrated greater improvements in glycemic control with combined exercise than with either modality alone in individuals with type 2 diabetes (3, 4). Consistent with these findings, a Bayesian network meta-analysis conducted by Liu et al. showed that 14–48 weeks of combined aerobic and resistance exercise produced the greatest reductions in hemoglobin A1c among individuals with type 2 diabetes. Of note, meaningful reductions in hemoglobin A1c (~0.75%) were already evident after 2–12 weeks of combined exercise training, whereas comparable short-term benefits were not observed with aerobic or resistance exercise in isolation. Although greater overall energy expenditure may partly explain these findings, the meta-analysis by Wang et al., further demonstrated that combined exercise training was associated with a greater improvement in vascular function (assessed by arterial stiffness and flow-mediated dilation), compared with resistance exercise alone. Given the close links between vascular function, insulin resistance, and oxidative stress (5, 6), the vascular adaptations associated with exercise interventions may represent an additional mechanism contributing to improved glycemic control.

Exercise may also influence the development of diabetic complications. Diabetic kidney disease, a common microvascular complication, affects more than 30% of the individuals with diabetes (7). Despite the well-established association between increased physical activity and reduced risks of cardiovascular events among individuals with diabetes (8), evidence linking exercise to diabetic kidney disease has been limited. In a multicenter cross-sectional study involving participants with diabetes from 40 healthcare facilities, Liu et al. found that achieving guideline-recommended amount of leisure-time physical activity, including both aerobic and resistance exercise, was associated with lower odds of diabetic kidney disease. In contrast, greater amounts of household and occupational physical activity were associated with higher odds for diabetic kidney disease. However, these findings should be interpreted cautiously given the cross-sectional study design, which precludes causal inference. Future studies using a randomized controlled design are warranted to determine whether structured exercise intervention can prevent the onset or progression of diabetic kidney disease, in particular in the real-world settings.

Collectively, the studies in this Research Topic have strengthened evidence supporting exercise as a key component for diabetes prevention and management. However, there remains the challenge on how exercise interventions could be optimally implemented in clinical practice. Key questions include whether structured exercise programs provide clinically meaningful benefits beyond standard exercise advice when long-term adherence is considered (9), and how exercise can be integrated with complementary lifestyle interventions, such as time-restricted eating, to further improve glycemic control while preserving physical function (10, 11). Addressing these questions will require pragmatic clinical trials that better reflect real-world populations and implementation challenges.

Finally, this Research Topic introduces the concept of ‘ExerScience’. While the established term ‘Exercise is Medicine’ primarily frames exercise or physical activity as a therapeutic prescription, “ExerScience’ advocates a broader and more integrative paradigm. It encompasses the continuum from molecular and cellular adaptations to population-level health outcomes, bridging basic science, clinical practice, and public health. Although the studies presented in this Research Topic predominantly evaluate clinical efficacy rather than underlying mechanisms, they collectively highlight the growing maturity of exercise science in diabetes research. We hope this Research Topic stimulates further multidisciplinary investigations that will establish ExerScience as a comprehensive framework for advancing personalized diabetes prevention and care.
